# Preclinical HistoBench: A Pilot Benchmark Dataset for Evaluating Large Language Models on Preclinical Histopathological Classification

**DOI:** 10.3390/biology15050395

**Published:** 2026-02-27

**Authors:** Avan Kader, Marie-Luise H. H. Ranner-Hafferl, Felix Reuter, Miriam L. Fichtner, Marcus R. Makowski, Keno K. Bressem, Lisa C. Adams

**Affiliations:** 1 Department of Diagnostic and Interventional Radiology, Technical University of Munich, Ismaninger Str. 22, 81675 Munich, Germany; felix.reuter@tum.de (F.R.); marcus.makowski@tum.de (M.R.M.); keno.bressem@tum.de (K.K.B.); 2 Department of Radiology, Charité–Universitätsmedizin Berlin, Corporate Member of Freie Universität Berlin, Humboldt-Universität zu Berlin, Charitéplatz 1, 10117 Berlin, Germany; marie.ranner-hafferl@charite.de; 3 Department of Experimental Neurology and Neurology, Charité–Universitätsmedizin Berlin, Corporate Member of Freie Universität Berlin, Humboldt-Universität zu Berlin, Charitéplatz 1, 10117 Berlin, Germany; miriam.fichtner@charite.de; 4 Department of Cardiovascular Radiology and Nuclear Medicine, Technical University of Munich, Ismaninger Str. 22, 81675 Munich, Germany

**Keywords:** benchmark dataset, preclinical histopathology, large language models, multi-task classification, digital animal pathology

## Abstract

**Background and Purpose**: We present a pilot benchmark dataset of 378 preclinical histological samples for evaluating large language model (LLM) performance on multi-dimensional classification tasks. This dataset addresses the lack of standardized benchmarks for assessing LLMs in preclinical histopathology, encompassing species identification (mouse, rabbit, rat), organ recognition, staining methods, and preparation techniques. **Methods**: We evaluated the LLMs GPT-4.1, GPT-4o-mini, and Llama 3.2 on 378 histological samples across four classification dimensions: species identification (mouse, rabbit, rat), organ recognition (kidney, liver, prostate, spleen), staining method classification (H&E, Elastica van Gieson, collagen, iron, IHC-elastin, MOVAT’s pentachrome), and preparation type determination (frozen vs. paraffin-embedded). Performance was assessed using sensitivity and specificity metrics with confusion matrix analysis. **Results**: Model performance varied substantially across tasks and exhibited strong sensitivity to class imbalance. For preparation type classification, GPT-4.1 achieved the most balanced performance (50% frozen sensitivity, 85.7% paraffin sensitivity), while Llama 3.2 failed to recognize paraffin samples (0% sensitivity). In species classification, Llama 3.2 was the only model capable of identifying all three species (rabbit: 75% sensitivity, rat: 85.7% sensitivity) despite poor mouse recognition (0.3% sensitivity). GPT-4.1 achieved higher mouse sensitivity within this dataset (70.4% sensitivity) but failed with minority species. For staining classification, Llama 3.2 demonstrated highest overall performance, achieving >88% sensitivity for most staining types, while GPT-4o-mini showed perfect H&E recognition (100% sensitivity). **Conclusions**: Current LLMs demonstrate variable performance for histological classification with substantial sensitivity to class imbalance. While not suitable for standalone diagnostic use, they may serve as useful screening tools in research settings with appropriate human oversight.

## Simple Summary

This study evaluates the capability of large language models to perform multi-dimensional classification of preclinical histological samples, addressing the absence of standardized benchmarks in this domain. We assessed three language models (GPT-4.1, GPT-4o-mini, and Llama 3.2) using 378 histological samples across four classification dimensions: species identification (mouse, rabbit, rat), organ recognition (kidney, liver, prostate, spleen), staining method classification (including H&E and specialized stains), and preparation technique determination (frozen versus paraffin-embedded). Our findings reveal substantial variability in model performance across tasks, with pronounced sensitivity to class imbalance. GPT-4.1 demonstrated superior performance for mouse identification (70.4% sensitivity) but failed to recognize minority species, while Llama 3.2 uniquely identified all three species despite poor mouse recognition. For staining classification, Llama 3.2 achieved the highest overall performance with greater than 88% sensitivity for most staining types. Preparation type classification proved particularly challenging, with only GPT-4.1 achieving balanced recognition of both frozen and paraffin-embedded samples. These results indicate that current large language models lack the reliability required for standalone diagnostic applications in histopathology. However, they may serve as valuable preliminary screening tools in research environments when combined with expert validation, potentially accelerating workflow efficiency while maintaining diagnostic accuracy through human oversight.

## 1. Introduction

Histopathological examination is the cornerstone of diagnostic medicine and biomedical research, providing important insights into tissue architecture, cell morphology, and the pathogenesis of diseases [1]. Accurate interpretation of histological sections requires extensive expertise in recognizing different tissue types, identifying specific staining patterns, and distinguishing between different species and preparation methods. This specialized knowledge demands extensive training and experience, with significant inter-observer variability reported even among expert pathologists, as individual interpretation can vary substantially based on experience, training background, and subjective assessment criteria [2,3]. The time-intensive nature of manual histological analysis and the need for specialized expertise remain significant challenges, despite advances in digital pathology infrastructure, particularly when large cohorts require comprehensive multi-dimensional tissue characterization across species, organ types, and staining protocols.

Recent developments in artificial intelligence and computer vision have shown promising applications in medical imaging, with deep learning algorithms achieving notable performance in specific pathological classification tasks under controlled conditions [4,5,6,7,8]. Large language models (LLMs), initially developed for natural language processing, have recently shown remarkable versatility in multimodal applications, including medical image analysis (X-rays, MRIs, and CT scans) [9]. Contemporary LLMs such as GPT-4 and its variants have demonstrated unprecedented capabilities in interpreting complex visual information and generating relevant responses [10,11,12,13,14]. These capabilities present particular opportunities for preclinical research settings, where automated classification systems could significantly enhance the efficiency of animal studies by enabling rapid identification of species, tissue types, and experimental conditions from histological samples, thereby reducing manual assessment time and improving standardization across research protocols. The potential clinical, veterinary medicine and animal research implications of automated histological classification systems are substantial. In clinical pathology laboratories, such tools could serve as intelligent screening systems, reducing diagnostic turnaround-times and providing quality assurance through consistent preliminary assessments [15,16]. For research applications, automated classification could enable large-scale tissue phenotyping, facilitate biobank organization, and support high-throughput studies requiring standardized histological analysis [17]. However, their specific application to histopathological image classification, particularly for simultaneous multi-attribute analysis encompassing species identification, tissue type recognition, staining method determination, and preparation technique classification, remains largely unexplored.

The primary contribution of this study is the development of a standardized pilot benchmark dataset and multi-dimensional evaluation framework for assessing LLM performance in preclinical histopathology. Using this framework, we evaluate three representative LLM architectures (Llama 3.2, GPT-4o-mini, and GPT-4.1) across multiple histological classification dimensions to establish baseline performance metrics. Our analysis encompasses species identification (mouse, rabbit, rat), organ type recognition (kidney, liver, prostate, spleen), histochemical staining classification (hematoxylin and eosin, Elastica van Gieson (elastin), Picrosirus red (collagen), Perls Prussian Blue (iron), immunohistochemical elastin, MOVAT’s pentachrome), and preparation method determination (frozen versus paraffin-embedded sections). This study aims to answer three key questions: (1) Can current-generation LLMs accurately classify preclinical histology images across multiple dimensions? (2) How do different LLM architectures compare in handling severe class imbalance inherent in real-world datasets? (3) What is the minimum performance threshold required for these models to provide practical utility in research settings?

## 2. Materials and Methods

### 2.1. Dataset Collection and Preparation

A total of 378 histological samples were collected from preclinical studies (Table 1) [18,19,20,21,22,23,24]. The dataset comprised tissue samples from three species: mouse (*n* = 367), rabbit (*n* = 4), and rat (*n* = 7), representing four organ types: prostate (*n* = 367), liver (*n* = 6), kidney (*n* = 3), and spleen (*n* = 2). Tissue preparation methods included frozen sections (*n* = 364) and paraffin-embedded sections (*n* = 14).

Histochemical staining was performed using six different methods: Collagen/Picrosirius red (*n* = 69), Elastica van Gieson (*n* = 207), Hematoxylin and Eosin (H&E) (*n* = 61), immunohistochemical elastin staining (*n* = 3), iron/Perls Prussian Blue (*n* = 21) and MOVAT’s pentachrome (*n* = 17), and All tissue sections were digitized using Keyence microscope (BZ-X800 Series, Keyence, Osaka, Japan). The quantification of the probes was measured with the image analysis software BZ-X800 Analyzer, version 1.1.30.19 (Keyence, Osaka, Japan).

### 2.2. Image Preprocessing

Digital histological images underwent standardized preprocessing to ensure consistency of the dataset. Quality control measures included independent visual assessment by two experienced reviewers: the first reviewer had several years of experience in histological analysis, and the second independent reviewer had expertise in histopathological assessment. Both reviewers independently assessed image quality and sample suitability to ensure that only suitable samples appropriate for computer-assisted analysis were included. A total of 400 histological images were initially collected. Image quality was assessed using a standardized five-point quality scale evaluating sharpness, cellular differentiation, fiber visibility, and exposure level for each staining type independently. Only images rated 3 or above were included in the final dataset. A total of 22 mouse prostate samples were excluded due to poor image quality (rated 1–2), resulting in the final benchmark dataset of 378 images. All rabbit and rat samples met quality criteria and were included without exclusion. Images were acquired using the Keyence BZ-X800 microscope at magnifications of 10×, 20×, and 40×, depending on the staining protocol and tissue type. The dataset comprises both single-frame acquisitions and representative patches from larger tissue sections. All images were saved in JPEG format without rescaling. Labels for species, organ, staining method, and preparation type were known from the original experimental protocols and did not require independent annotation. Image quality was independently assessed by two reviewers using the standardized five-point quality scale described above. The mean score across both reviewers was calculated for each image, and images with a mean score below 3.0 were excluded from the final dataset.

### 2.3. Classification Tasks and Evaluation Framework

Four distinct classification tasks were defined:

*Preparation Type Classification:* Models distinguished between frozen and paraffin-embedded tissue sections based on morphological artifacts and tissue preservation characteristics.

*Species Classification:* Models were required to identify tissue origin as mouse, rabbit, or rat based on histological features visible in the digitized sections.

*Organ Classification:* Models classified tissue samples into four organ categories: kidney, liver, prostate, and spleen, based on characteristic histological architecture and cellular organization.

*Staining Classification:* Models identified the histochemical staining method used, including H&E, Elastica van Gieson, Collagen/Picrosirius red, Iron/Perls Prussian Blue, IHC-elastin, and MOVAT’s pentachrome, based on characteristic color patterns and tissue contrast.

### 2.4. Model Selection

We selected three representative LLM architectures to evaluate different approaches to multimodal image analysis. GPT-4.1 (OpenAI) represents the current state-of-the-art in commercial multimodal models with advanced vision capabilities. GPT-4o-mini (OpenAI) was included as a cost-effective alternative to assess whether smaller, optimized models could achieve comparable performance. Llama 3.2 (Meta) was selected as an open-source alternative suitable for institutions with data privacy requirements. Specifically, we used Llama-3.2-90B-Vision-Instruct-Turbo (meta-llama/Llama-3.2-90B-Vision-Instruct-Turbo), the 90 billion parameter instruction-tuned vision-language variant, accessed through the Together AI inference API. This model can alternatively be deployed locally on appropriate hardware (e.g., NVIDIA H100 GPUs with vLLM) for complete data isolation when processing sensitive data.

### 2.5. Prompt Engineering

We iteratively developed our classification prompt through systematic testing of five different prompt formats, evaluating each for consistency and accuracy on a validation subset of 20 images. The final prompt was structured to provide clear categorical options while avoiding leading language that might bias model responses. We specifically included all possible categories upfront to prevent models from defaulting to majority classes and emphasized that only one answer should be selected per category to ensure consistent output formatting.

The system prompt established the model’s role: “You are a histology expert. You are given a histology slide image and a question about it. You will answer the question based on the image.” The user prompt provided the image and specified the classification task: “Here is a histology slide image. Please identify the staining type, the animal species, the preparation type, and the tissue type.” followed by explicit enumeration of all valid categories for each dimension (staining: H&E, Collagen, MOVAT, IHC-Elastin, Iron, Elastica van Gieson; species: Mouse, Rat, Rabbit; preparation: Frozen, Paraffin; tissue: Prostate, Kidney, Spleen, Liver). For OpenAI models, outputs were constrained through structured parsing using Pydantic (version 2.8.2) with enumerated categories enforced at the API level. For Llama 3.2, explicit JSON formatting instructions were appended to the prompt requiring the model to return only a valid JSON object with the four classification fields. The complete prompts and Pydantic schema definitions are provided in the Supplementary Materials (Supplementary Materials, S1, S2 and S3).

### 2.6. Model Inference and Evaluation

We evaluated three vision-capable LLM in a zero-shot, closed-set setting. Each image was processed independently. A concise system prompt established histology expertise and a single user prompt listed the allowed labels for staining, species, preparation, and tissue as defined above. Outputs were constrained through an ontology-aligned schema with one categorical field per dimension, implemented via structured parsing using Pydantic (version 2.8.2). The models returned exactly one label per dimension. No fine-tuning, few-shot examples, rationales, or auxiliary text were used.

Images were supplied as JPEGs without rescaling to standardize input handling. The specific model versions used were: GPT-4.1 (gpt-4.1-2025-04-14) and GPT-4o-mini (gpt-4o-mini-2025-04-16) accessed via OpenAI Responses API with schema parsing, and Llama 3.2 (meta-llama/Llama-3.2-90B-Vision-Instruct-Turbo) accessed via Together AI inference API. All three models used temperature = 0 to ensure deterministic, reproducible outputs and eliminate sampling variance. No post-processing beyond schema enforcement was applied. Each model produced one prediction per image and predictions were stored per image for downstream analysis.

For each dimension, predictions were compared against reference labels to construct confusion matrices. Class-wise sensitivity and specificity were computed in a one-versus-rest manner. Each image took approximately 3–5 s to process using GPT-4.1, 2–3 s using GPT-4o-mini, and 1–2 s using Llama 3.2via Together AI. Processing times varied based on image complexity and API response latency.

### 2.7. Statistical Analysis

We selected sensitivity and specificity as primary evaluation metrics because they provide interpretable class-specific performance measures essential for understanding model behavior under severe class imbalance, where aggregate metrics such as accuracy would be dominated by majority class performance and mask critical failures in minority class recognition. Sensitivity directly addresses whether a model can identify samples of a given class when present, while specificity addresses false positive rates. The binary one-versus-rest calculation framework enables consistent comparison across classification dimensions with varying numbers of classes (2 classes for preparation type, 3 for species, 4 for organs, 6 for staining). Sensitivity and specificity were calculated using standard formulas from confusion matrices generated for each model’s predictions. Sensitivity was defined as true positives divided by the sum of true positives and false negatives, while specificity was defined as true negatives divided by the sum of true negatives and false positives. All calculations were performed using Python 3.11 with NumPy and Scikit-learn libraries. Due to the extreme class imbalance in our dataset, we did not perform McNemar’s test or other comparative statistics, as these would be unreliable with such small minority class samples. *p*-values were not calculated due to insufficient sample sizes in minority classes. No correction for multiple comparisons was applied. Future studies with balanced datasets should incorporate more comprehensive statistical analyses including confidence intervals and formal hypothesis testing.

All experiments used fixed random seeds (seed = 42) for reproducible results. All three models (GPT-4.1, GPT-4o-mini, and Llama 3.2) used temperature = 0 to eliminate sampling variance and ensure deterministic outputs.

Due to the binary nature of our classification tasks and the severe class imbalance in our dataset, confidence intervals were not calculated for individual sensitivity and specificity values.

## 3. Results

We evaluated three LLMs on our preclinical histopathology pilot benchmark dataset to establish baseline performance metrics for future comparisons.

### 3.1. Preparation Type Classification Performance

**Figure 1:**
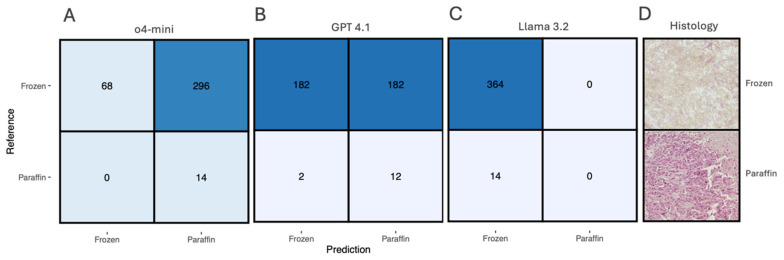
Comparative performance of large language models for preparation type classification. Confusion matrices showing the classification performance of (**A**) GPT-4o-mini, (**B**) GPT-4.1, and (**C**) Llama 3.2 for distinguishing between frozen and paraffin-embedded tissue sections. Numbers in each cell represent the count of samples, with rows indicating reference (true) labels and columns showing predicted labels. Blue shading intensity corresponds to the numerical values, with darker shading indicating higher counts. (**D**) Representative histological examples showing characteristic morphological differences between frozen sections (top: showing crystalline artifacts) and paraffin-embedded sections (bottom: displaying uniform tissue preservation). The dataset comprised 378 total samples: 364 frozen sections and 14 paraffin-embedded sections.

The classification of tissue preparation methods (frozen vs. paraffin-embedded sections) revealed substantial differences in performance across the three LLM architectures (Figure 1). The dataset comprised 378 samples, with 364 frozen sections and 14 paraffin-embedded sections.

#### Model Performance Comparison

*GPT-4o-mini* showed a strong bias toward paraffin classification, achieving 100% paraffin but only 18.7% frozen sensitivity (Figure 1A). *GPT-4.1* demonstrated the most balanced performance, with 50% frozen and 85.7% paraffin sensitivity (Figure 1B). *Llama 3.2* classified all samples as frozen, achieving 100% frozen but 0% paraffin sensitivity (Figure 1C).

In summary, GPT-4.1 achieved the most balanced performance across both preparation types, followed by GPT-4o-mini, while Llama 3.2 showed complete failure to recognize paraffin-embedded sections.

### 3.2. Species Classification Performance

**Figure 2:**
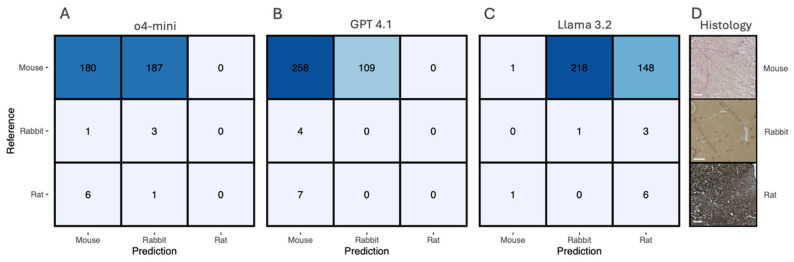
Comparative performance of large language models for species classification. Confusion matrices showing the classification performance of (**A**) GPT-4o-mini, (**B**) GPT-4.1, and (**C**) Llama 3.2 for distinguishing between mouse, rabbit, and rat tissue samples. Numbers in each cell represent the count of samples, with rows indicating reference (true) labels and columns showing predicted labels. Blue shading intensity corresponds to the numerical values, with darker shading indicating higher counts. (**D**) Representative histological examples showing tissue samples from each species: mouse (**top**), rabbit (**middle**), and rat (**bottom**). The dataset comprised 378 total samples with marked class imbalance: 367 mouse samples, 4 rabbit samples, and 7 rat samples.

Species classification across mouse, rabbit, and rat samples showed marked performance differences between the three LLM architectures (Figure 2). The dataset contained 378 samples with substantial class imbalance: 367 mouse samples, 4 rabbit samples, and 7 rat samples.

#### Model Performance Comparison

*GPT-4o-mini* achieved 49.0% mouse and 75.0% rabbit sensitivity but failed to identify rat samples (Figure 2A). *GPT-4.1* showed the highest mouse sensitivity (70.3%) but classified all minority species as mouse (Figure 2B). *Llama 3.2* was the only model identifying all three species, though with very low mouse sensitivity (0.3%) and higher sensitivity for rabbit (75.0%) and rat (85.7%) (Figure 2C). It should be noted that rabbit (*n* = 4) and rat (*n* = 7) sample sizes are too small to draw robust conclusions. The reported sensitivities for these classes should be interpreted as illustrative rather than definitive.

It should be noted that rabbit (*n* = 4) and rat (*n* = 7) sample sizes are too small to draw robust conclusions about model performance for these species. The reported sensitivities for these classes should be interpreted as illustrative rather than definitive.

In summary, Llama 3.2 was the only model capable of identifying all three species, showing superior performance for minority classes. GPT-4o-mini demonstrated moderate performance across mouse and rabbit classification, while GPT-4.1 achieved the highest mouse sensitivity but failed completely with minority species.

### 3.3. Organ Classification Performance

**Figure 3:**
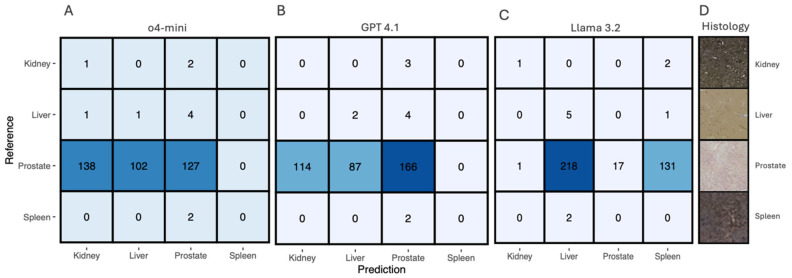
Comparative performance of large language models for organ classification. Confusion matrices showing the classification performance of (**A**) GPT-4o-mini, (**B**) GPT-4.1, and (**C**) Llama 3.2 for distinguishing between kidney, liver, prostate, and spleen tissue samples. Numbers in each cell represent the count of samples, with rows indicating reference (true) labels and columns showing predicted labels. Blue shading intensity corresponds to the numerical values, with darker shading indicating higher counts. (**D**) Representative histological examples showing tissue samples from each organ type: kidney (**top**), liver (**second**), prostate (**third**), and spleen (**bottom**). The dataset comprised 378 total samples with extreme class imbalance: 367 prostate samples, 6 liver samples, 3 kidney samples, and 2 spleen samples.

Organ classification across kidney, liver, prostate, and spleen samples revealed distinct performance patterns among the three LLM architectures (Figure 3). The dataset contained 378 samples with pronounced class imbalance: 367 prostate samples, 6 liver samples, 3 kidney samples, and 2 spleen samples.

#### Model Performance Comparison

*GPT-4o-mini* showed limited recognition across all organ types, with the highest sensitivity for prostate (34.6%) (Figure 3A). *GPT-4.1* achieved the highest prostate sensitivity (45.2%) but failed to identify kidney and spleen samples (Figure 3B). *Llama 3.2* showed the highest liver sensitivity (83.3%) but poor prostate recognition (4.6%) (Figure 3C). Given the very small sample sizes for kidney (*n* = 3), liver (*n* = 6), and spleen (*n* = 2), the reported sensitivities for these organ types are illustrative and should not be interpreted as robust performance estimates. All models failed to identify spleen samples correctly. Given the very small sample sizes for kidney (*n* = 3), liver (*n* = 6), and spleen (*n* = 2), the reported sensitivities for these organ types are illustrative and should not be interpreted as robust performance estimates.

In summary, GPT-4.1 achieved the highest prostate sensitivity within this dataset, Llama 3.2 achieved the highest liver sensitivity within this dataset, while GPT-4.1 showed moderate liver recognition. All models failed to identify spleen samples correctly.

### 3.4. Staining Classification Performance

**Figure 4:**
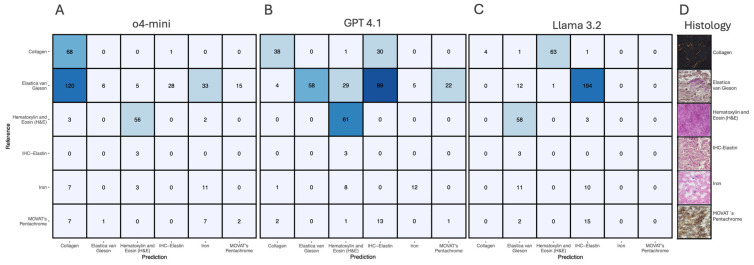
Comparative performance of large language models for staining classification. Confusion matrices showing the classification performance of (**A**) GPT-4o-mini, (**B**) GPT-4.1, and (**C**) Llama 3.2 for distinguishing between six histochemical staining methods: Collagen, Elastica van Gieson, Hematoxylin and Eosin (H&E), IHC-Elastin, Iron, and MOVAT’s Pentachrome. Numbers in each cell represent the count of samples, with rows indicating reference (true) labels and columns showing predicted labels. Blue shading intensity corresponds to the numerical values, with darker shading indicating higher counts. (**D**) Representative histological examples showing characteristic staining patterns for each method: Picrosirus red—Collagen (**top**), Elastica van Gieson- Elastin (**second**), Hematoxylin and Eosin—H&E (**third**), IHC-Elastin (**fourth**), Perls Prussian Blue Iron (**fifth**), and MOVAT’s Pentachrome (**bottom**). The dataset comprised 378 total stainings with class imbalance: 207 Elastica van Gieson, 69 Collagen, 61 H&E, 21 Iron, 17 MOVAT’s Pentachrome, and 3 IHC-Elastin samples.

Staining method classification across six histochemical techniques showed variable performance among the three LLM (Figure 4). The dataset contained samples with substantial class imbalance: 207 Elastica van Gieson (Elastin) samples, 69 Picrosirus red (collagen) samples, 61 Hematoxylin and Eosin (H&E) samples, 21 Perls Prussian Blue (Iron) samples, 17 MOVAT’s Pentachrome samples, and 3 IHC-Elastin samples.

#### Model Performance Comparison

*GPT-4o-mini* achieved near-perfect collagen detection (98.6%) and good H&E recognition (91.8%), but showed poor sensitivity for Elastica van Gieson (2.9%) and MOVAT’s Pentachrome (11.8%) (Figure 4A). *GPT-4.1* achieved perfect H&E recognition (100%) and moderate performance for collagen (55.1%) and iron (57.1%), but showed poor sensitivity for Elastica van Gieson (28.0%) and MOVAT’s Pentachrome (5.9%) (Figure 4B). *Llama 3.2* achieved the highest overall staining sensitivity, with strong recognition across most staining types (93.7% Elastica van Gieson, 95.1% H&E, 88.2% MOVAT’s Pentachrome), but poor Collagen recognition (5.8%) (Figure 4C). The IHC-Elastin results (*n* = 3) should be interpreted with particular caution given the minimal sample size. All models failed to identify IHC-Elastin samples correctly. The IHC-Elastin results (*n* = 3) should be interpreted with particular caution given the minimal sample size.

In summary, Llama 3.2 achieved the highest overall staining sensitivity within this dataset with strong recognition across most staining types (93.7% Elastica van Gieson, 95.1% H&E, 88.2% MOVAT’s Pentachrome, 100% IHC-Elastin), followed by GPT-4o-mini with nearly perfect collagen detection (98.6%). GPT-4.1 achieved perfect H&E recognition (100%) but showed more variable performance across other staining methods.

## 4. Discussion

The Preclinical HistoBench pilot benchmark reveals three key insights about current LLM capabilities in histopathological classification: (1) extreme sensitivity to class imbalance, (2) model-specific failure modes, and (3) complementary strengths suggesting ensemble potential. These baseline results establish performance standards for future model development.

For preparation type classification, GPT-4.1 demonstrated the most balanced performance between frozen and paraffin-embedded sections, while Llama 3.2 exhibited complete failure to recognize paraffin samples. These findings are consistent with previous research by Gorman et al., who demonstrated that models trained on frozen sections performed well when tested on other slide preparations, but models trained on only formalin-fixed tissue performed significantly worse across other modalities [25]. The fundamental difference in tissue preparation artifacts may contribute to these observed differences in model performance, with frozen sections showing crystalline structures while FFPE sections display smoother morphology [26]. This technical distinction appears to be differentially recognized by the three model architectures, suggesting varying sensitivity to morphological artifacts introduced during tissue processing.

Species classification presented unique challenges, with each model displaying distinct failure modes. GPT-4o-mini showed moderate capability for recognizing rabbit samples despite their low representation, while GPT-4.1 achieved higher mouse sensitivity within this dataset but failed completely with minority species. Llama 3.2 was the only model capable of identifying all three species, though with very low accuracy in mouse detection. These differential recognition patterns reflect species-specific histological characteristics documented in comparative anatomy studies. Mouse tissue morphology has been extensively characterized in terms of cellular organization and structural features [27,28], while rat tissue architecture shows distinct organizational patterns that differ from other rodent species [29,30]. Rabbit tissues demonstrate unique morphological characteristics, including differences in mesenchymal stem cell size and tissue organization compared to smaller rodent models [31,32,33]. These morphological differences represent distinct visual features that may be differentially recognized by different LLM architectures based on their underlying pattern recognition capabilities.

The extreme class imbalances present in our dataset (96.3% frozen vs. 3.7% paraffin, 97.1% mouse samples) significantly influenced model behavior, with all models showing tendency to over-classify toward dominant classes, except for Llama 3.2 which demonstrated different behavior in species classification. This bias toward majority classes is a well-documented limitation in machine learning applications, as most learners exhibit bias towards the majority class and in extreme cases may ignore the minority class altogether [34]. The ability of Llama 3.2 to maintain sensitivity for minority species (75% for rabbit, 85.7% for rat) despite severe class imbalance “suggests potentially higher sensitivity for minority classes within this dataset, though this came at the cost of reduced sensitivity for the majority class, highlighting the fundamental challenge of achieving balanced performance across all classes in severely imbalanced datasets. This represents a critical challenge for deploying these systems in real-world scenarios where rare conditions or sample types must be accurately identified.

The staining classification results revealed interesting patterns in how different models recognize histochemical techniques, with GPT-4.1 perfect recognition of H&E staining (100% sensitivity) likely reflecting the prevalence of H&E-stained images in general medical image datasets, as H&E is the most commonly used staining method in diagnostic pathology. Conversely, Llama 3.2 demonstrated strong performance across most staining types, achieving 95.1% sensitivity for H&E and 93.7% for Elastica van Gieson, suggesting effective recognition of both standard and specialized histochemical staining patterns. However, its poor Collagen recognition (5.8%) remains unexplained and warrants further investigation. The consistent failure across all models to identify IHC-elastin samples (0% sensitivity) may reflect both the small sample size (*n* = 3) and the technical complexity of immunohistochemical staining interpretation, as IHC requires recognition of specific protein localization patterns rather than general tissue morphology, representing a more specialized diagnostic task that may exceed current LLM capabilities.

The variable performance across different classification tasks has important implications for potential clinical and research applications, though the inconsistent performance patterns suggest that current LLM technology may not yet be reliable enough for standalone diagnostic applications. However, several potential use cases emerge from our findings, particularly in research settings where these models could serve as screening tools or assist in organizing large histological datasets. The ability of certain models to identify minority classes suggests potential utility in biobank organization and sample sorting, with Llama 3.2’s capability to identify rare species being valuable for quality control in multi-species studies, flagging potentially mislabeled samples for human review. Models showing high sensitivity for specific staining methods could serve as initial screening tools to categorize large histological datasets, reducing manual workload for pathologists and researchers. The complementary strengths of different models suggest potential for ensemble approaches, where multiple models could be used in combination to improve overall classification accuracy and reliability.

Our results contribute to the understanding of LLM applications in digital pathology, particularly for preclinical histopathological classification tasks. While recent advances in deep learning have shown promising results for specific clinical pathological classification tasks [35,36], the variable performance observed across different classification dimensions in our study suggests that significant challenges remain for reliable implementation of LLMs in specialized medical contexts, despite showing promise for certain applications. Based on our findings, several priorities emerge for future development of LLM-based histopathological classification systems. Development of training strategies and loss functions specifically designed to handle extreme class imbalances common in medical datasets is crucial, with techniques such as focal loss, oversampling of minority classes, or specialized ensemble methods potentially improving performance for rare sample types. The complementary strengths observed across different models suggest that ensemble methods combining multiple LLMs could achieve more balanced and reliable performance than any single model. Future work should explore the development of LLMs specifically trained on preclinical histopathological datasets, potentially improving performance compared to general-purpose models fine-tuned for preclinical applications. The creation of larger, more balanced benchmark datasets would facilitate more robust model comparisons and support the development of more reliable AI tools for histopathological analysis.

### 4.1. Statistical Limitations and Baseline Considerations

The small sample sizes in minority classes (*n* = 4 rabbits, *n* = 2 spleens, *n* = 3 kidneys) preclude formal hypothesis testing for comparative performance claims. Our descriptive sensitivity and specificity metrics should be interpreted as preliminary estimates rather than definitive performance benchmarks. We intentionally avoided calculating *p*-values and confidence intervals given insufficient statistical power for reliable inference. Claims about relative model performance (e.g., “Llama 3.2 demonstrated higher minority class sensitivity within this dataset”) reflect observed patterns in this specific dataset and require validation on larger, independent samples before generalization. A majority-class baseline classifier would achieve high overall accuracy (96.3% for preparation type, 97.1% for organ classification) by always predicting the dominant class, but with 0% sensitivity for all minority classes. The ability of tested LLMs to identify minority classes—despite modest overall performance—represents meaningful improvement over such naive baselines and demonstrates task-relevant pattern recognition beyond dataset priors. This study establishes a methodological framework and initial performance estimates to guide future adequately powered evaluations. The absence of CNN or pathology-specific model comparisons in this study reflects our specific focus on establishing baseline LLM capabilities in a zero-shot setting without fine-tuning or task-specific training. Such supervised models would require labeled training data from our specific classification tasks and cannot operate in the zero-shot paradigm we evaluated. They represent fundamentally different deployment scenarios: immediate applicability without training data (our LLM approach) versus optimized performance after task-specific training (CNN/specialized approaches). Future work should include comparisons with fine-tuned vision models to establish performance ceilings for these tasks and determine whether zero-shot LLMs can approach supervised model performance, which would have important implications for rapid deployment in new research contexts without extensive training data collection.

### 4.2. Limitations

This study has several limitations that should be considered when interpreting the results. The class imbalances in our dataset may not represent typical distributions encountered in research settings. The small sample sizes for minority classes also limit the statistical power for evaluating model performance on these categories. These limitations may affect the generalizability of our findings to more balanced datasets. Our evaluation used a single prompt configuration per model, validated on a small subset (*n* = 20 images). While this prompt demonstrated consistent performance during initial validation, we cannot fully disentangle model capability from prompt-specific effects. Systematic prompt robustness analyses across multiple formulations and decoding strategies would strengthen future evaluations by establishing performance stability across different prompting approaches. However, our focus on zero-shot performance with standardized prompts provides a reproducible baseline that future studies can build upon. Future work could address these limitations through several approaches. Data augmentation strategies could help balance class representations during training. Exploring ensemble methods that combine the strengths of different models may improve overall classification performance. The creation of larger, more balanced benchmark datasets would facilitate more robust model comparisons and support the development of more reliable AI tools for histopathological analysis.

The class imbalances in Preclinical HistoBench reflect the specific research focus of a single laboratory rather than general preclinical distributions. The predominance of mouse prostate samples (97.1%) results from the laboratory’s focus on prostate cancer research and limits generalization to other organ systems and species. While these imbalances represent realistic conditions within specialized research settings, they do not reflect the broader diversity of samples encountered in multi-center preclinical workflows. Future versions of this benchmark should include samples from multiple laboratories and research areas to improve generalizability.

## 5. Conclusions

The primary contribution of this study is a standardized pilot benchmark dataset and evaluation framework for assessing LLM performance in preclinical histopathology. The documented class imbalances and performance baselines establish reference points for future model development, rather than providing definitive comparisons between current architectures. Future applications of this pilot benchmark include evaluation of fine-tuned models specifically trained on preclinical histopathological data, systematic testing of ensemble approaches combining multiple LLM architectures, and assessment of imbalance-aware training strategies such as focal loss or class-specific oversampling. The standardized evaluation framework established here provides a reproducible foundation for these future investigations.

## Figures and Tables

**Table 1:** Dataset composition showing distribution of samples across classification categories.

Category	Subcategory	Number of Samples	Percentage
Species			
	Mouse	367	97.1%
	Rabbit	4	1.1%
	Rat	7	1.8%
Preparation Type			
	Frozen	364	96.3%
	Paraffin-embedded	14	3.7%
Organ			
	Prostate	367	97.1%
	Liver	6	1.6%
	Kidney	3	0.8%
	Spleen	2	0.5%
Staining Method			
	Elastica van Gieson	207	54.8%
	Collagen	69	18.3%
	H&E	61	16.1%
	Iron	21	5.6%
	MOVAT’s pentachrome	17	4.5%
	ICH-Elastin	3	0.3%
Total		378	100%

## Data Availability

The datasets used and/or analysed during the current study are available from the corresponding author on reasonable request.

## Supplementary Materials

The following supporting information can be downloaded at: https://www.mdpi.com/article/10.3390/biology15050395/s1, S1: Promt Configurations, S1.1: System Prompt, S1.2: User Prompt (OpenAI Models), S1.3: User Promt (Llama 3.2), S2: Pydantic Schema for Structured Output; S3: API Configuration, S3.1: OpenAI API Call, S3.2: Together AI API Call (Llama 3.2).

## Abbreviations

The following abbreviations are used in this manuscript:

LLM	large language model
H&E	hermatoxylin and eosin
