# Supplementary material for: Preclinical HistoBench: A Pilot Benchmark Dataset for Evaluating Large Language Models on Preclinical Histopathological Classification
Source: Biology (Basel). 2026 Feb 27;15(5):395. doi: 10.3390/biology15050395 (PMC12984232; doi:10.3390/biology15050395)
Supplement: Supplementary file 1 [file biology-15-00395-s001.zip › biology-4112039-supplementary.pdf]

Supplementary Materials

PreclinicalHistoBench: A Pilot Benchmark Dataset for Evaluating Large Language Models on Preclinical Histopathological Classification

S1. Prompt Configuration

S1.1 System Prompt

The following system prompt was used for all models to establish the histology expert role:

```
You are a histology expert. You are given a histology slide image and a
question about it. You will answer the question based on the image.
```

S1.2 User Prompt (OpenAI Models)

The following user prompt was used for GPT-4.1 and GPT-4o-mini, with structured output enforced via Pydantic schema:

```
Here is a histology slide image. Please identify the staining type, the
animal species, the preparation type, and the tissue type.  Possible staining
types are: - Hematoxylin and Eosin (H&E) - Collagen - MOVAT (MOVAT's
Pentachrome) - IHC-Elastin - Iron - Elastica van Gieson  Possible animal
species are: - Mouse - Rat - Rabbit  Possible preparation types are: - Frozen
- Paraffin  Possible tissue types are: - Prostate - Kidney - Spleen - Liver
Here is the image:
```

S1.3 User Prompt (Llama 3.2)

For Llama 3.2, the user prompt included additional JSON formatting instructions since the Together AI API does not support native structured output:

```
Here is a histology slide image. Please identify the staining type, the
animal species, the preparation type, and the tissue type.  Possible staining
types are: - Hematoxylin and Eosin (H&E) - Collagen - MOVAT (MOVAT's
Pentachrome) - IHC-Elastin - Iron - Elastica van Gieson  Possible animal
species are: - Mouse - Rat - Rabbit  Possible preparation types are: - Frozen
- Paraffin  Possible tissue types are: - Prostate - Kidney - Spleen - Liver
ANSWER ONLY IN THIS FORMAT:  {     "staining_type": "",     "animal_species":
"",     "preparation_type": "",     "tissue_type": "" }  DO ONLY RETURN A
VALID JSON. Here is the image:
```

S2. Pydantic Schema for Structured Output

The following Pydantic schema (version 2.8.2) was used to enforce structured output for OpenAI models via the Responses API:

```
from enum import Enum from pydantic import BaseModel  class
StainingType(Enum):     H_E = "Hematoxylin and Eosin (H&E)"     COLLAGEN =
"Collagen"     MOVAT = "MOVAT's Pentachrome"     IHC_ELASTIN = "IHC-Elastin"
IRON = "Iron"     ELASTICA_VAN_GIESON = "Elastica van Gieson"  class
```

```
AnimalSpecies(Enum):     MOUSE = "Mouse"     RAT = "Rat"     RABBIT =
"Rabbit"  class PreparationType(Enum):     FROZEN = "Frozen"     PARAFFIN =
"Paraffin"  class TissueType(Enum):     PROSTATE = "Prostate"     KIDNEY =
"Kidney"     SPLEEN = "Spleen"     LIVER = "Liver"  class
SlideClassification(BaseModel):     staining_type: StainingType
animal_species: AnimalSpecies     preparation_type: PreparationType
tissue_type: TissueType
```

S3. API Configuration

S3.1 OpenAI API Call

```
response = client.responses.parse(     model=model,  # "gpt-4.1-2025-04-14"
or "gpt-4o-mini-2025-04-16"     input=input,     temperature=0.0,
text_format=SlideClassification, )
```

S3.2 Together AI API Call (Llama 3.2)

```
response = client.chat.completions.create(     model="meta-llama/Llama-3.2-
90B-Vision-Instruct-Turbo",     messages=input,     temperature=0.0,
stream=False )
```
